# Elucidation of drought tolerance mechanisms of different mungbean genotypes based upon physiological, biochemical and genetic mechanisms

**DOI:** 10.3389/fpls.2026.1710212

**Published:** 2026-03-04

**Authors:** Fahad M. Alghabari

**Affiliations:** Department of Agriculture, Faculty of Environmental Sciences, King Abdulaziz University, Jeddah, Saudi Arabia

**Keywords:** antioxidant, correlation, gene regulation, heatmap, osmoprotectants, PCA

## Abstract

Drought is a major problem to mungbean (*Vigna radiata* L.) productivity, necessitating the identification of tolerant genotypes and the exploration of their adaptive mechanisms. This study evaluated seven mungbean genotypes ‘BARI Mung-8’, ‘BMX-010015’, ‘K851’, ‘L-92’, ‘BARI Mung-1’, ‘FH-18’, and ‘PDM-139’ under control and drought treatments to characterize their physiological, biochemical, and molecular responses. Physiological traits, including chlorophyll content, photosynthesis rate (Pn), cell membrane stability (CMS), and relative water content (RWC), varied significantly (p≤ 0.05). Under drought, ‘BARI Mung-8’, ‘BMX-010015’, and ‘K851’ maintained chl content of 1.85–2.10 mg g^-1^ FW and Pn of 138–145 μmol m^-2^ s^-1^, compared to 1.25 mg g^-1^ FW and 78 μmol m^-2^ s^-1^ in ‘BARI Mung-1’. These tolerant lines also retained high RWC (89–92%) and CMS (84–86%). Biochemically, they accumulated greater osmolytes, proline (38.7–42.1 µg g^-1^ FW) and glycine betaine (118–132 µg g^-1^ FW), and depicted enhanced antioxidant enzyme activities, including SOD (39.8–41.2 U mg^-1^ protein) and CAT (14.5–15.2 U mg^-1^ protein). Principal component analysis (PCA) and heatmap clustering grouped tolerant genotypes with these key adaptive traits, illustrating combined stress-response processes. Gene expression profiling showed significant upregulation (2.5–4.8 fold) of osmotic adjustment genes (*VrP5CS1*, *VrBADH*), antioxidant defense genes (*VrSOD1*, *VrCAT1*, *VrPOD1*), water transport gene (*VrPIP2-1*), and stress signaling genes (*VrDREB2A*, *VrLEA3*). The aquaporin gene *VrPIP2–1* was associated with higher RWC, while *VrCHLH* stability supported chl retention. Integration of physiological, biochemical, and molecular data proved that drought tolerance in mungbean is regulated by coordinated cellular hydration, osmotic regulation, ROS detoxification, and transcriptional activation. “BARI Mung-8’, ‘BMX-010015’, ‘K851’, and ‘L-92’ emerged as eminent candidates for breeding programs targeting drought-prone environments, and the identified genes provide potential markers for selection of genotypes in climate-resilient legume improvement.

## 1 Introduction

Drought stands as an imminent challenge to world agriculture, particularly in semi-arid and arid regions, where water deficiency severely limits crop yield (Islam et al., 2023). With the increasing impacts of climate change, the intensity and frequency of drought events are expected to increase, posing significant threats to global food security (Ali et al., 2025). Legume crops, including mungbean (*Vigna radiata* L.), are especially vulnerable to water scarcity, which can disrupt critical growth stages such as germination, flowering, and pod filling, causing substantial yield reductions (Kumar et al., 2021).

Mungbean is a warm-season short duration legume crop widely cultivated in South and Southeast Asia (Ahn and Shanmugasundaram, 2019). It is important for its rapid growth cycle, high protein content, and ability to fix atmospheric nitrogen, thereby enhancing soil fertility (Ahmad et al., 2024). Despite these advantages, mungbean’s sensitivity to drought stress reduces its productivity, particularly under rainfed conditions prevalent in various arable regions (Islam et al., 2023). Addressing this challenge requires a broad understanding of the mechanisms inducing drought tolerance in mungbean to support the breeding programs aimed at developing resilient cultivars (Chang et al., 2023).

Besides, plants have developed various adaptive responses to cope with drought stress, causing morphological, physiological, biochemical, and molecular changes (Ali et al., 2025). Physiologically, drought stress often leads to reduced leaf water, stomatal closure, and decreased photosynthetic rates (Singh et al., 2021). Biochemically, drought escalates the accumulation of osmoprotectants such as proline, glycine betaine and soluble sugars, which maintain cell turgor and protect cellular structures (Chaudhary et al., 2022). In addition, drought stress triggers the production of reactive oxygen species (ROS), leading to oxidative damage (Ahmad et al., 2024). To mitigate this, plants increase the activity of antioxidant enzymes like superoxide dismutase (SOD), catalase (CAT), and peroxidase (POD) to scavenge ROS and protect cellular integrity (Ighodaro and Akinloye, 2018).

At the molecular level, drought stress triggers complex signaling pathways that control the expression of stress-responsive genes (Kumar et al., 2021). In mungbean (*Vigna radiata*), various key genes play critical role in drought tolerance. For instance, *VrP5CS1* (*Δ¹-pyrroline-5-carboxylate synthetase 1*) and *VrBADH* (*Betaine aldehyde dehydrogenase*) are respectively involved in the synthesis of osmoprotectants proline and glycine betaine, both compounds maintain osmotic balance and protect cellular structures under water stress (Raina et al., 2016; Singh et al., 2021). The oxidative defense system comprises *VrSOD1* (*Superoxide dismutase 1*), *VrCAT1* (*Catalase 1*), and *VrPOD1* (*Peroxidase 1*), where *VrSOD1* detoxifies superoxide-radicals into hydrogen peroxide (H_2_O_2_), and *VrCAT1* and *VrPOD1* further dissociates the hydrogen peroxide (H_2_O_2_), thus protecting cells from oxidative damage during drought stress (Ariharasutharsan et al., 2022). Moreover, *VrPIP2-1* (*Plasma membrane intrinsic protein 2-1*), an aquaporin gene, assists water transport across membrane, keeping water homeostasis under water deficit conditions (Al-Dulaimi et al., 2022). As a whole, these genes boost the tolerance of mung bean to drought stress through osmotic adjustment, oxidative stress mitigation, and efficient water regulation.

Despite these advancements, there is lack of integrated studies that unanimously assess physiological, biochemical, and genetic responses to drought stress across diverse mungbean genotypes (Alsamadany, 2022). Such comprehensive researches are vital for elucidating the complicated interplay of traits that impart drought tolerance and for identifying superior genotypes for cultivation in water-deficit environments (Islam et al., 2023).

In this context, the present study aimed to elucidate the drought tolerance mechanisms of different mungbean genotypes by evaluating a set of physiological parameters (e.g., relative water content, chlorophyll content) (Raina et al., 2016), biochemical markers (e.g., proline accumulation, antioxidant enzyme activities) (Ahmad et al., 2024), and the expression profiles of key drought-responsive genes (Alsamadany, 2022). By combining the data from these traits, we intend to identify genotypes with high drought tolerance and to unravel the underlying physiological and molecular mechanisms that impart this tolerance (Kumar et al., 2021). This study will facilitate the breeding programs to develop mungbean varieties that will grow well in drought, ensuring more food in dry areas.

## 2 Materials and methods

In the present study, mungbean germplasm (**Table 1**) including drought tolerant cultivars and susceptible was sourced from research institutions of Pakistan, Uzbekistan, Bangladesh and India. The genotypes assessed for drought tolerance through physiological, biochemical, and molecular indicators. The experiment followed a two-factorial design, considering mungbean genotypes as the first factor and treatment conditions (control and salinity stress) as the second. A pot trial with three replicates was executed under a randomized complete block design (RCBD) at the research facility of King Abdulaziz University, Jeddah, Saudi Arabia (21°32′36″N, 39°10′22″E), situated 12m above the level of sea.

**Table 1:** List of drought tolerant and susceptible mung bean genotypes evaluated in study.

Mungbean cultivars	Country	Reference
Drought Tolerant		
L-92	Uzbekistan	Azimov et al., 2024
K851	India	Kumar et al., 2020
BMX-010015	Bangladesh	Islam et al., 2021
BARI Mung-8	Bangladesh	Islam et al., 2023
Drought Susceptible		
BARI Mung-1	Bangladesh	Islam et al., 2023
FH-18	Pakistan	Islam et al., 2023
PDM-139	India	Kumar, 2023

### 2.1 Plant material growth and drought stress imposition

Certified seeds of mungbean genotypes were surface-sterilized using 2% sodium hypochlorite solution for 3 minutes, followed by continuous rinsing with double distilled water. The seeds were sown in plastic pots with 25 cm diameter having a mixture of loamy soil, sand, and farmyard manure in a 2:1:1 ratio (v/v). The mixture was prepared to ensure proper aeration and drainage. Pots were kept under controlled greenhouse conditions, with regular irrigation to support uniform germination and seedling. For each treatment 5 pots, each with five plants were used. When seedlings attained the two to three leaf stage approximately 15–20 days after sowing, the drought stress was applied by withholding irrigation until the soil moisture content reduced to 30–40% of field capacity, calculated using the gravimetric method, following the method explained by Kumar et al. (2020). Besides, control plants were irrigated daily to maintain 80–100% field capacity. Drought stress was maintained progressively till the appearance of drought symptoms (Singh et al., 2021). During the stress period, plants were monitored for physiological, biochemical and genetic responses.

### 2.2 Enzymatic antioxidant activity

The activity of key antioxidant enzymes was measured spectrophotometrically, using the protocols outlined by Alici and Arabaci (2016). For this purpose, freshly detached mungbean leaves were washed with distilled water and 10 g of finely chopped leaf tissue was homogenized in 50 mL of 100 mM sodium phosphate buffer (pH 7.0) containing 1 mM ascorbic acid and 0.5% (w/v) polyvinylpyrrolidone (PVP). The homogenate was incubated using buffer system for 5 minutes at 4 °C before filtration through muslin cloth. The filtrate was centrifuged (5000 × g) for 5 minutes at and the resulting supernatant was used to execute enzymatic assays. Catalase (CAT) activity was estimated by monitoring the decrease in absorbance at 240 nm due to the breakdown of hydrogen peroxide (H_2_O_2_) at room temperature. Peroxidase (POD) activity was measured by finding the increase in absorbance at 420 nm, using 4-methylcatechol as the substrate. The activity of superoxide dismutase (SOD) was calculated based on its ability to inhibit the photoreduction of nitroblue tetrazolium (NBT). Furthermore, the reaction mixture was exposed to white light (10–15 minutes), to record absorbance at 560 nm. Besides, the enzymatic activity was expressed in enzyme units, mg-1 of protein.

### 2.3 Estimation of glycine betaine and proline

Glycine betaine (GB) content from mungbean leaves was measured by Grieve and Grattan (1983) method. Fresh leaf samples of mungbean cultivars were homogenized in deionized water, and the extract was mixed thoroughly with H_2_SO_4_ (2N) and potassium iodide-iodine (KI-I_2_) reagent. The mixture was centrifuged at 3000 × g for approximately 5 minutes. Subsequently, dichloroethane was mixed to the supernatant, and the absorbance of the resulting pink-colored phase was recorded at 365 nm by spectrophotometer (Mettler Toledo, USA). Proline concentration was determined as per the protocol of Bates et al. (1973). Fresh leaves from mungbean cultivars were homogenized in 3% sulfosalicylic acid, and the extract was reacted with acidic ninhydrin and glacial acetic acid. The reaction mixture was heated at 80 °C for 1 hour, and followed by extraction with toluene. The absorbance of the chromophore with toluene layer was measured at 520 nm using a spectrophotometer (Mettler Toledo, USA).

### 2.4 Measurement of physiological traits

Key physiological parameters of mungbean genotypes including photosynthetic rate (Pn), were measured from fully expanded leaves using a portable Infrared Gas Analyzer (IRGA; ADC Bioscientific, UK). Besides, the total chlorophyll content of mungbean leaves was measured non-destructively under glasshouse conditions using a special apparatus, SPAD-502 Plus Chlorophyll Meter (Konica Minolta, Korea). Healthy and fully expanded leaves from the third trifoliate node were selected from each plant for chl quantification. For consistency, readings were recorded between 09:00 and 11:00 AM under controlled glasshouse light conditions. For this purpose, the sensor probe of the SPAD meter was gently clamped on the leaf lamina, avoiding the midrib, and five readings per leaf were recorded at different positions and averaged. Besides, the assessment of cell membrane stability percentage (CMSP) was done by evaluating electrolyte leakage from leaf tissues, following the relative conductivity method proposed by Ibrahim and Quick (2001). After thorough and continuous washing, the leaf samples were dipped in vials having 10 mL of deionized water and incubated at 10 °C for 18 hours. Subsequently, the samples were maintained at room temperature for 1 hour. To support electrolytic diffusion, the samples were further incubated at 10 °C for 24 hours. After this period, the samples were equilibrated at room temperature, shaken, and the initial value of electrical conductivity (E1) was recorded using an Odeon conductivity meter (Aqualabo, France). Subsequently, the samples were autoclaved at 121 °C and 0.10 MPa for 15 minutes to release total electrolytes. After cooling and shaking, the final value of electrical conductivity (E2) was measured. The CMSP was calculated by the formula:


CMSP = [1 − (E1/E2)] × 100.

### 2.5 RNA extraction and analysis of gene expression

For gene expression analysis, mungbean plants were sampled at the appearance of initial drought stress symptoms. Collected leaf samples were immediately frozen in liquid nitrogen container and preserved at −80 °C until the start of RNA extraction. Total RNA was isolated using the RNeasy Plant Mini Kit (Qiagen, Germany) as per the manufacturer’s protocol. Subsequently, 2 µg of total RNA was utilized for cDNA synthesis using the QuantiTect Reverse Transcription Kit (Qiagen, Germany). The expression patterns of drought-responsive genes including *VrDREB2A*, *VrP5CS1, VrPIP2-1, VrCAT1, VrPOD1, VrSOD1, VrCHLH, VrLEA3 and VrBADH* were analyzed via quantitative real-time PCR (qRT-PCR) using SYBR Green PCR Master Mix (BioFACT, Korea). Besides, the expression was normalized using the mungbean housekeeping gene *VrActin1*. Each qRT-PCR reaction was performed in three technical and three biological replicates, following the methodology opted by Zhou et al. (2023). The relative quantification of gene expression was calculated using the 2^−ΔΔCt method. The list of primers used is provided in **Table 2**.

**Table 2:** List of gene primers used for relative expression analysis.

Gene	Primers	Accession no
VrDREB2A	ATGGCGGAGGAAGAGATGGA (F) TCACTTGGCTGGGAGATCCT (R)	MW698173.1
VrP5CS1	ATGACCGAGGAGATGGAGAA (F) TGGTCACCTTGGTGAACTTG (R)	MW396576.1
VrPIP2-1	GCTGTGCTTCTTGCTCTTCC (F) GGACAGCAAGCAGAGACCTT(R)	MN123699.1
VrCAT1	AGGAGCTGCTGAGGTAGGTG (F) GGCTTGATGGTGGTGAACTC (R)	KY862375.1
VrPOD1	TCTCGTGGACTTCGAGATGG (F) GTTGGTGAGGTCCTTGTTGG	KY962414.1
VrSOD1	CTTGTCACCGTCTGCAAGAA (F) GGTCCTTGGTGGTGGTGAAT (R)	KY962415.1
VrCHLH	TGAGGCTGCTGAGGTAGTTG (F) ATGATGGAGCCGTTGAACTG (R)	OM001355.1
VrLEA3	GCTTCTTCCTCAGCTCTTCC (F) CCGTCTTGGTGATGTTGAGG (R)	MW362614.1
VrBADH	ATGGAGGAGAAGGCGGAGAA (F) TCACTTGGTGGAGGTGGTTG (R)	KY965848.1

### 2.6 Statistical analysis

Data were analyzed using analysis of variance (ANOVA) in computer based Statistix software version 8.1 (Statistix, 2003), applying a 5% level of significance. Furthermore, visualization of data, such as correlation matrices, principal component analysis (PCA) biplots, and hierarchical clustering heatmaps, was carried out using RStudio version 1.1.456 (RStudio Team, 2023). The PCA was executed using the “factoextra” and “FactoMineR” R packages, while Pearson’s correlation analysis was performed with the “GGally” and “ggplot2” packages. Heatmap generation and dendrogram construction were performed using the “pheatmap” and “ComplexHeatmap” packages in R.

## 3 Results

### 3.1 Physiological traits

**Figure 1:**
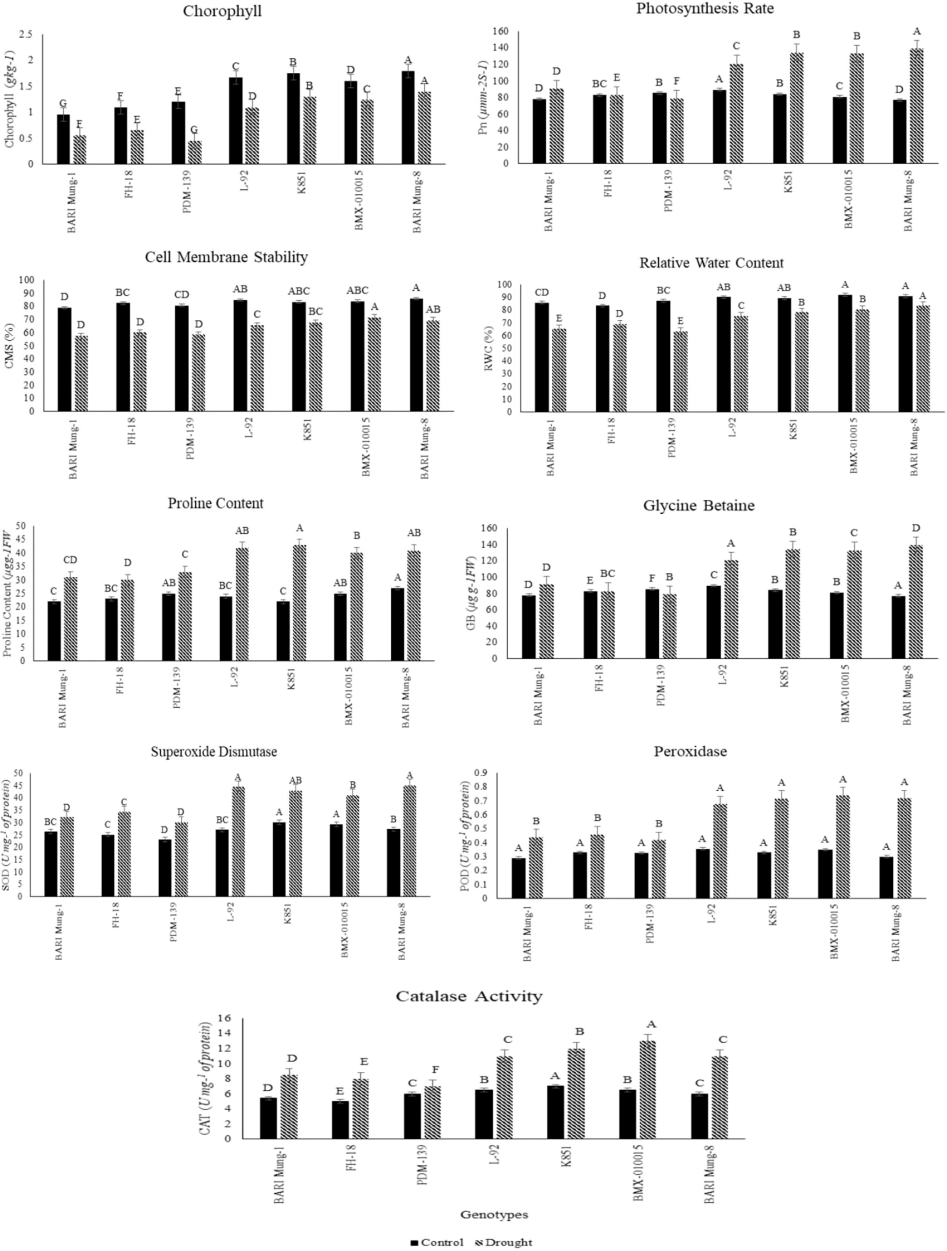
Physiological, osmoprotectant, and antioxidant enzyme responses of drought tolerant and susceptible mungbean genotypes under control and drought conditions. The traits include chlorophyll content, Pn, photosynthesis rate; CMS, cell membrane stability; RWC, relative water content; proline content; GB, glycine betaine; SOD, superoxide dismutase; POD, peroxidase; CAT, catalase (CAT) activities. Data are indicated as mean ± SE (n = 3), and different letters represents statistically significant differences among genotypes within each treatment according to *post hoc* tests at p < 0.05 during tri-replicate experiment.

Four physiological traits including chlorophyll content (chl), photosynthesis rate (Pn), cell membrane stability (CMS), and relative water content (RWC) illustrated the significant variation (p ≤ 0.05) among seven mungbean genotypes under control and drought conditions (**Figure 1**). Chlorophyll content (chl) varied significantly, with tolerant genotypes like ‘BARI Mung-8’ and ‘BMX-010015’ maintaining higher values under drought (2.10 and 1.85 mg g^-1^ FW, respectively), whereas susceptible genotypes such as ‘BARI Mung-1’ and ‘FH-18’ showed much lower values (1.25 and 1.40 mg g^-1^ FW) (**Figure 1**). Photosynthesis rate (Pn) also varied significantly under drought, where ‘BARI Mung-8’ recorded the highest rate (145 μmol m^-2^ s^-1^) followed by ‘K851’ (138 μmol m^-2^ s^-1^), while ‘BARI Mung-1’ and ‘PDM-139’ exhibited the lowest values (78 and 85 μmol m^-2^ s^-1^, respectively) (**Figure 1**). CMS differed significantly across genotypes, with ‘BARI Mung-8’ and ‘BMX-010015’ showing higher membrane stability under drought (86% and 84%, respectively), while susceptible lines like ‘BARI Mung-1’ and ‘FH-18’ had significantly lower CMS values (62% and 68%) as indicated in **Figure 1**. Similarly, RWC also showed significant differences; tolerant genotypes such as ‘BARI Mung-8’, ‘BMX-010015’, and ‘K851’ retained higher RWC under drought (92%, 90%, and 89%, respectively), whereas ‘BARI Mung-1’ and ‘FH-18’ dropped to 68% and 71% (**Figure 1**). These findings proved that all four physiological traits were significantly affected by drought stress at the 0.05 probability level (p ≤ 0.05), with tolerant genotypes consistently performing better than susceptible ones, indicating their superior drought resilience.

### 3.2 Biochemical traits

The key biochemical traits proline content, glycine betaine (GB), superoxide dismutase (SOD), peroxidase (POD), and catalase (CAT) demonstrated the significant variation (p≤ 0.05) among seven mungbean genotypes under control and drought stress conditions (**Figure 1**). Proline content varied significantly across genotypes, with drought-tolerant genotypes such as ‘K851’ (42.1 µg g^-1^ FW), ‘BARI Mung-8’ (40.3 µg g^-1^), and ‘BMX-010015’ (38.7 µg g^-1^) showing the highest accumulation under drought, while susceptible genotypes like ‘BARI Mung-1’ and ‘FH-18’ recorded significantly lower values (26.4 and 28.1 µg g^-1^) (**Figure 1**). Similarly, glycine betaine (GB) content also increased significantly under drought, with ‘K851’ (132 µg g^-1^ FW), ‘L-92’ (120 µg g^-1^), and ‘BARI Mung-8’ (118 µg g^-1^) exhibiting higher concentrations, compared to lower levels in ‘PDM-139’ (98 µg g^-1^) and ‘FH-18’ (87 µg g^-1^) as illustrated in **Figure 1**. Antioxidant enzyme activities also varied significantly. SOD activity was the highest in ‘K851’ (41.2 U mg^-1^ protein) and ‘L-92’ (39.8 U mg^-1^), while the lowest values were recorded in ‘PDM-139’ (27.3 U mg^-1^) and ‘FH-18’ (29.5 U mg^-1^) (**Figure 1**). POD activity showed less genotypic variation but still increased significantly under drought, with all genotypes clustering around 0.70–0.75 U mg^-1^ protein in tolerant lines, compared to 0.55–0.60 in susceptible ones (**Figure 1**). Catalase activity (CAT) showed the most distinct differences, with ‘BMX-010015’ and ‘K851’ recording the highest activities (15.2 and 14.5 U mg^-1^ protein), while ‘BARI Mung-1’ and ‘FH-18’ remained significantly lower (8.1 and 7.3 U mg^-1^, respectively) (**Figure 1**). These findings confirmed that drought-tolerant genotypes exhibit superior biochemical responses that align with their physiological performance under stress.

### 3.3 Correlation matrix, principal component and heatmap interpretations

**Figure 2:**
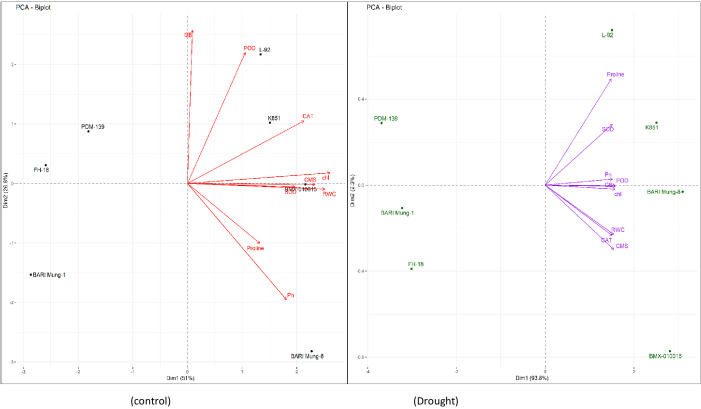
Principal component analysis (PCA) biplots showing the relationship between physiological, osmoprotectant and antioxidant enzyme traits, and mungbean genotypes under control (left) and drought (right) conditions. The traits include chlorophyll content, Pn, photosynthesis rate; CMS, cell membrane stability; RWC, relative water content; proline content, GB, glycine betaine; SOD, superoxide dismutase; POD, peroxidase; CAT, catalase activities. The separation of genotypes and trait vectors illustrates distinct clustering trends, with drought-tolerant mungbean genotypes aligning more strongly with drought-associated traits such as proline, GB, and antioxidant enzymes.

**Figure 3:**
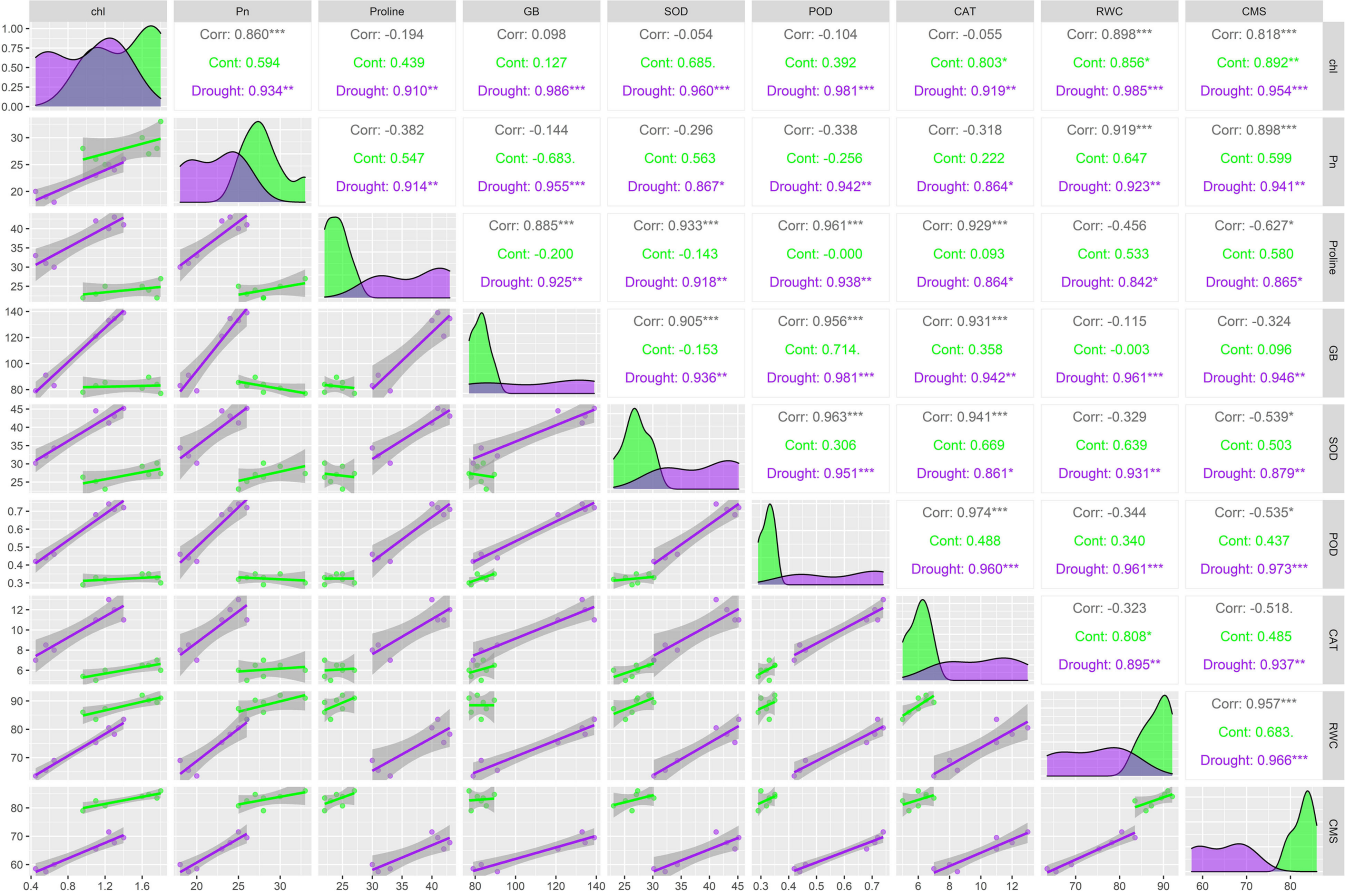
Pairwise correlation analysis among physiological, osmoprotectant and antioxidant enzymes traits of mungbean genotypes under control (green) and drought (purple) treatments. The traits include chlorophyll content, Pn, photosynthesis rate; CMS, cell membrane stability; RWC, relative water content; proline content, GB, glycine betaine; SOD, superoxide dismutase; POD, peroxidase; CAT, catalase activities. Scatter plots with regression lines, density plots, and correlation coefficients (Pearson’s r) represents pairwise trait associations. Significance levels: *p* < 0.05 (*), p < 0.01 (**), p < 0.001 (***).

The comparative performance of different mungbean cultivars under control and drought conditions is illustrated in **Figure 2**. Under drought stress, tolerant genotypes such as ‘BARI Mung-8’, ‘BMX-010015’, ‘K851’, and ‘L-92’ clustered closely with key stress-adaptive traits including proline, GB, SOD, CAT, CMS, and RWC, reflecting their integrated and efficient drought responses (**Figure 2**). In contrast, susceptible genotypes like ‘BARI Mung-1’, ‘FH-18’, and ‘PDM-139’ were positioned farther from these vectors, indicating weak associations with protective traits. Under control conditions, genotype separation was less distinct, confirming that drought stress amplified trait divergence (**Figure 2**). The correlation matrix further supported these patterns by showing strong and significant positive associations among the studied traits under drought (**Figure 3**). For example, chlorophyll content (chl) showed a highly significant correlation with Pn (r = 0.934), while osmolytes correlated positively with CMS and antioxidant enzymes, suggesting a coordinated stress defense network in tolerant genotypes.

**Figure 4:**
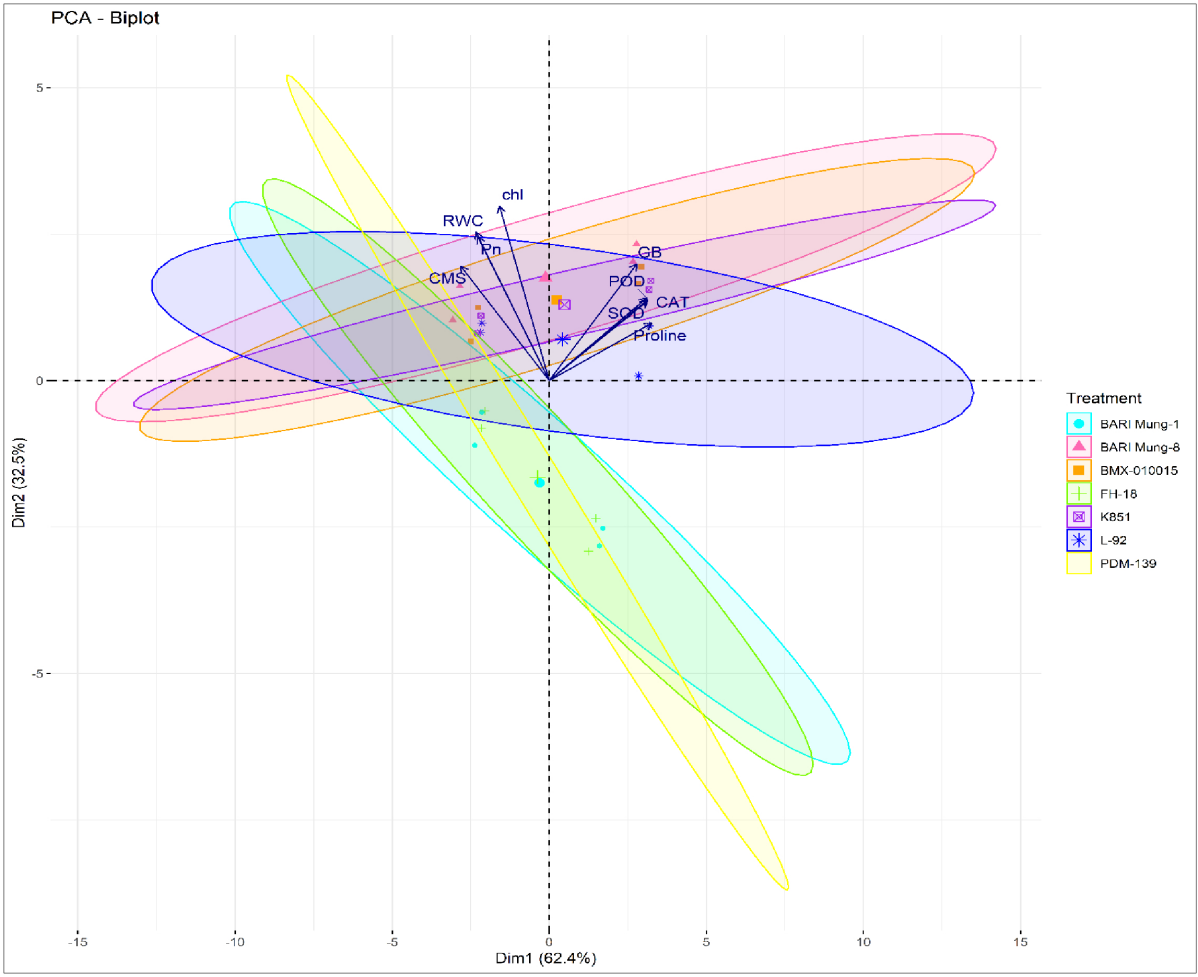
Principal component analysis (PCA) biplot illustrating the dispersion of ellipses of seven mungbean genotypes based on physiological, osmoprotectant and antioxidant traits under integrated drought and control datasets. The traits include chlorophyll content, Pn, photosynthesis rate; CMS, cell membrane stability; RWC, relative water content; proline content, GB, glycine betaine; SOD, superoxide dismutase; POD, peroxidase; CAT, catalase activities. Vectors indicate traits, and colored ellipses represent the 95% confidence intervals for each genotype.

**Figure 5:**
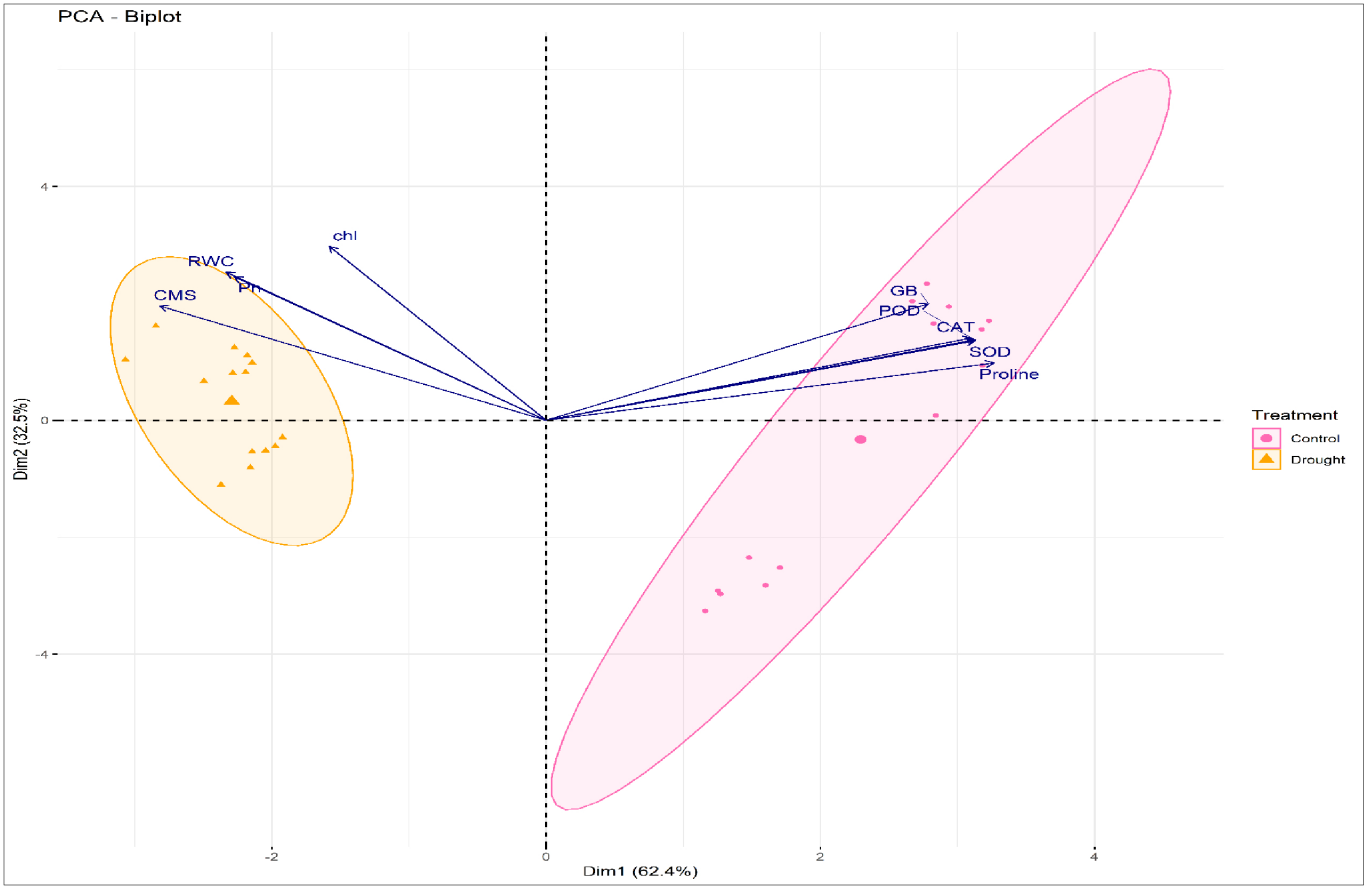
Principal component analysis (PCA) biplot indicating separation of control and drought treatments in mungbean genotypes based on physiological, osmoprotectant and antioxidant enzymes traits. The traits include chlorophyll content, Pn, photosynthesis rate; CMS, cell membrane stability; RWC, relative water content; proline content, GB, glycine betaine; SOD, superoxide dismutase; POD, peroxidase; CAT, catalase activities. Vectors represents the trait contribution, and ellipses represent 95% confidence intervals for each treatment group.

The multivariate analyses presented in **Figures 4** and **5** provided a comprehensive overview of trait interactions and cultivar performance under control and drought stress conditions. The PCA biplot (**Figure 4**) revealed clear ellipses separation between drought-tolerant and susceptible cultivars. Tolerant genotypes ‘BARI Mung-8’, ‘K851’, ‘L-92’, and ‘BMX-010015’ grouped closely with traits like proline, GB, CAT, SOD, RWC, and CMS, whereas susceptible genotypes ‘BARI Mung-1’, ‘FH-18’, and ‘PDM-139’ were distantly scattered and weakly associated with these stress-protective traits. This clustering pattern aligns with physiological and biochemical results, confirming that multivariate trait integration plays a central role in drought tolerance. Besides, the PCA biplot, with clear ellipses for each treatment, demonstrated distinct clustering of mungbean genotypes under control and drought treatments (**Figure 5**). The control group is closely associated with higher RWC, chl, Pn, and CMS, reflecting optimal physiological function. In contrast, the drought treated set of genotypes aligned with elevated proline, glycine betaine (GB), and antioxidant enzymes (SOD, CAT, POD), indicating the activation of drought-responsive biochemical mechanisms. This separation confirmed that drought stress significantly alters trait expression as compared to control treatments.

**Figure 6:**
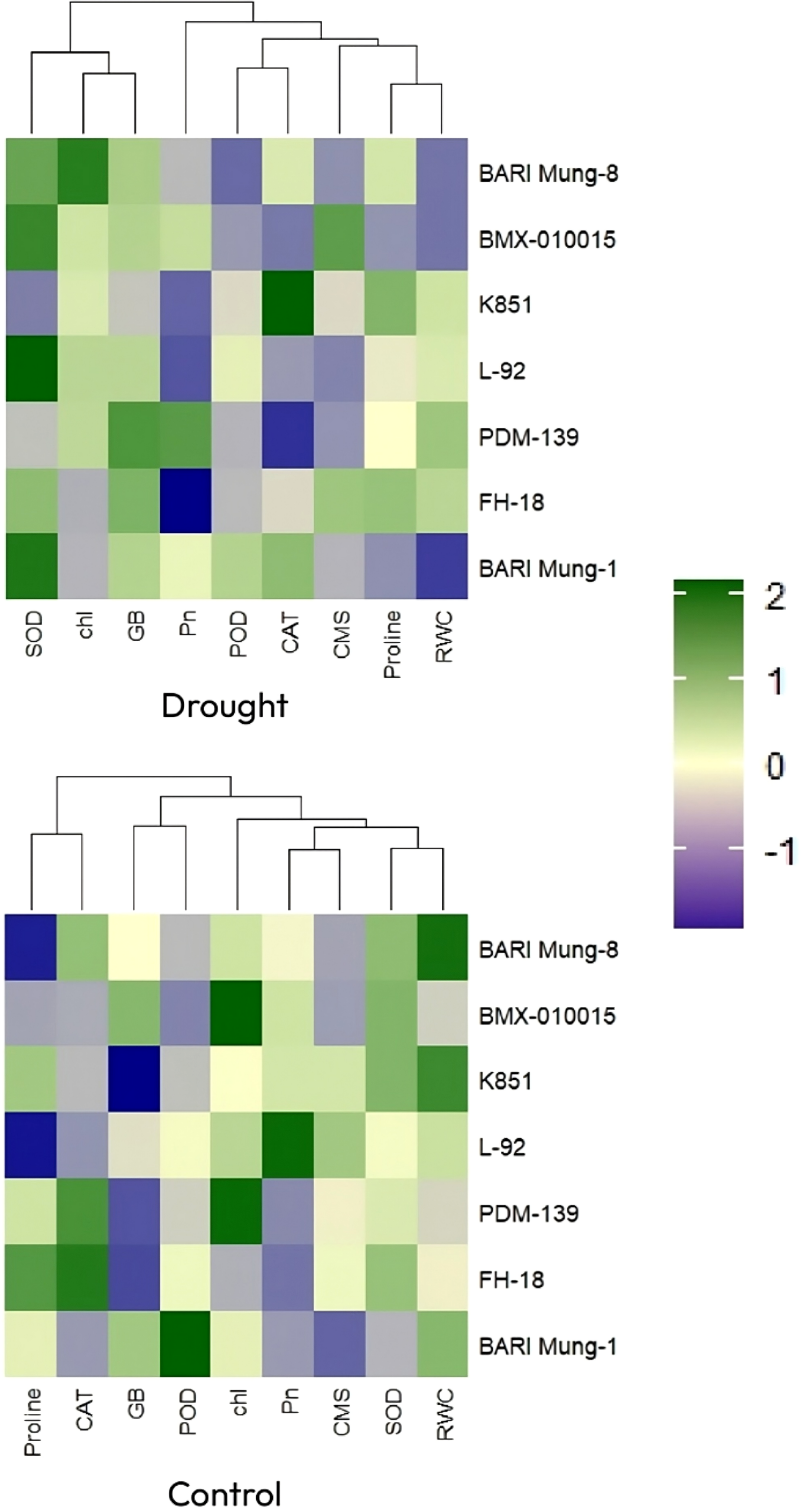
Hierarchical cluster heatmaps of physiological, osmoprotectant, and antioxidant enzymes traits of mungbean genotypes under control (bottom) and drought (top) conditions. The traits include chlorophyll content, Pn, photosynthesis rate; CMS, cell membrane stability; RWC, relative water content; proline content, GB, glycine betaine; SOD, superoxide dismutase; POD, peroxidase; CAT, catalase activities. Color intensity indicates standardized trait values (Z-scores), with dendrograms showing genotype clustering patterns.

Furthermore, the heatmap analysis showed how different mungbean genotypes responded to drought and normal conditions based on physiological and biochemical traits (**Figure 6**). Under water deficit condition, the drought-tolerant genotypes, **‘**BARI Mung-8’, ‘BMX-010015’, ‘K851’, and ‘L-92’ showed comparatively levels of adaptive traits like antioxidant enzymes (SOD, CAT, POD), proline, GB, RWC, and CMSP. These traits support the plants reduce damage and survive better during drought conditions. On the other hand, the drought-susceptible genotypes **‘**PDM-139’, ‘FH-18’, and ‘BARI Mung-1’ showed lower levels of these traits, indicating their weaker tendency to cope with drought stress. Although under normal conditions, the differences were smaller, but the tolerant genotypes still showed better overall performance. This heatmap analysis supports the findings from **Figures 2**-**5**, and further strengthens the associations shown in the correlation matrix and PCAs, confirming that physiological and biochemical traits co-regulate mungbean drought tolerance and reflect the combined effect of stress-responsive mechanisms.

### 3.4 Gene expression analysis

**Figure 7:**
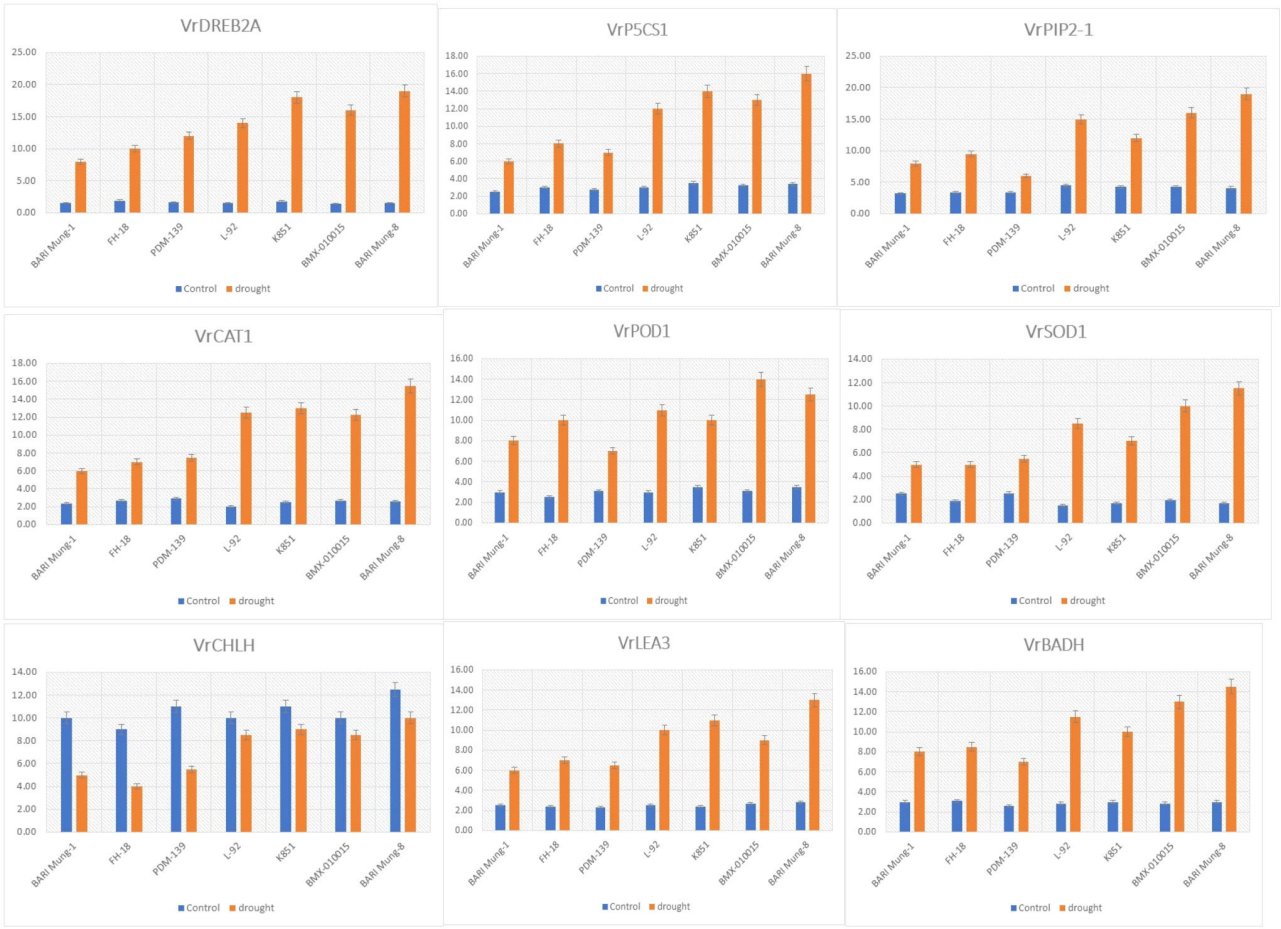
Relative expression patterns of drought-responsive genes in drought tolerant and susceptible mungbean genotypes under control (blue bars) and drought (orange bars) treatments. Genes analyzed include transcription factor *VrDREB2A*, osmolyte biosynthesis genes (*VrP5CS1*, *VrBADH*), water transport gene (*VrPIP2-1*), antioxidant defense genes (*VrCAT1*, *VrPOD1*, *VrSOD1*), chlorophyll biosynthesis gene (*VrCHLH*), and late embryogenesis abundant protein gene (*VrLEA3*). Values indicates means ± SE of three biological replicates.

The drought-responsive genes showed significant variation in expression among all mungbean genotypes under drought and control conditions (**Figure 7**). Overall, drought-tolerant genotypes ‘BARI Mung-8’, ‘BMX-010015’, ‘K851’, and ‘L-92’ consistently exhibited significantly higher expression of all tested genes under drought compared to the susceptible genotypes ‘BARI Mung-1’, ‘FH-18’, and ‘PDM-139’. Genes such as *VrDREB2A, VrP5CS1, VrLEA3*, and *VrBADH*, which are involved in stress signaling and osmolyte (proline and GB) synthesis, were strongly upregulated in tolerant genotypes, indicating enhanced drought adaptation mechanisms. Similarly, antioxidant-related genes *VrCAT1, VrSOD1*, and *VrPOD1* showed greater expression in tolerant genotypes, reflecting stronger protection against oxidative damage and directly corresponding to the increased CAT, SOD, and POD activities reported in **Figure 1**. Notably, *VrPIP2-1*, associated with water transport, showed significantly high expression in ‘BARI Mung-8’ and ‘L-92’, supporting their high RWC under drought, while *VrCHLH*, a gene involved in chlorophyll biosynthesis, was relatively stable in tolerant lines but downregulated in susceptible genotypes, matching the observed chlorophyll trends in **Figure 1**. These gene expression patterns further rectified the physiological and biochemical findings and provided strong molecular evidence that drought tolerance in mungbean is controlled by the coordinated upregulation of key stress-responsive genes that directly regulate osmotic adjustment, antioxidant activity, and water conservation.

## 4 Discussion

The present research provided an integrative understanding of drought tolerance mechanisms in mungbean by characterizing seven different genotypes using physiological, biochemical, multivariate, and molecular approaches. Drought stress caused a notable decline in chl, Pn, RWC, and CMSP across all genotypes (**Figure 1**). However, drought-tolerant genotypes such as ‘BARI Mung-8’, ‘BMX-010015’, and ‘K851’ exhibited higher values of these traits under drought, maintaining chl content between 1.85–2.10 mg g^-1^ FW and Pn above 130 µmol m^-2^ s^-1^, in contrast to values as low as 1.25 mg g^-1^ FW and 78 µmol m^-2^ s^-1^ in susceptible lines like ‘BARI Mung-1’. These physiological traits are key indicators of photosynthetic efficiency and drought stability, and their maintenance under stress explicates an active regulation of photoprotection and carbon assimilation processes. Notably, CMS and RWC values remained above 85% and 89%, respectively, in tolerant genotypes, indicating their ability for cellular hydration and membrane protection. These findings agree with the outcomes of Tiwari et al. (2023) and Singh et al. (2021), who reported similar trends of trait stability under drought stress in leguminous crops. The observed correlations among chl, Pn, RWC, and CMS (**Figure 3**) further affirmed that these physiological indicators are functionally linked and co-regulated during stress responses.

Biochemical adjustments also imparted a crucial role in drought tolerance. Drought-tolerant genotypes accumulated significantly higher levels of osmolytes, including proline and glycine betaine (GB), which increase osmotic regulation and cellular stability under water deficit conditions (**Figure 1**). For instance, ‘K851’ accumulated over 42 µg g^-1^ FW of proline and 132 µg g^-1^ FW of GB, substantially higher than the levels in susceptible genotypes. These osmolytes are widely reported as stress-protectants that stabilize proteins and membranes, prevent enzyme denaturation, and act as ROS scavengers as reported by Islam et al. (2023); Biswas et al. (2016). In parallel, tolerant genotypes exhibited higher antioxidant activities of SOD, POD, and CAT particularly ‘BMX-010015’ and ‘K851’, which had CAT activity as high as 15.2 U mg^-1^ protein. This enzymatic defense system protects oxidative damage by scavenging excess ROS produced during metabolic imbalance caused by water deficit stress, supporting findings from Ariharasutharsan et al. (2022); Nahar et al. (2015), and Mandi et al. (2018). The coordination among osmolyte levels and antioxidant enzymes activity was further affirmed by the strong positive correlations observed in **Figure 3**, where proline and GB were strongly associated with CMS and enzymatic activities, illustrating a system-wide metabolic adjustment to drought.

Multivariate analyses further proved these relationships and showed a robust picture of genotype-trait associations. PCA (**Figures 2**, **4**) clearly separated tolerant genotypes ‘BARI Mung-8’, ‘K851’, ‘BMX-010015’, and ‘L-92’ from susceptible ones, with tolerant genotypes clustering close to vectors representing proline, GB, RWC, CMS, SOD, and CAT. These findings align with past trait integration studies in pulses, such as those by Baroowa et al. (2016); Singh et al. (2021) and Islam et al. (2023), who reported synergistic trait networks as key regulators of drought tolerance. Besides, the PCA biplot (**Figure 5**) illustrated a clear separation between control and drought-treated mungbean genotypes, reflecting distinct adjustment approaches. Control plants were associated with higher RWC, chl, Pn, and CMS, representing normal physiological activity. Drought-treated plants manifested high proline, GB, and antioxidant enzymes (SOD, CAT, POD) activities, indicating osmotic adaptations and oxidative stress defense. These results are consistent with previous studies in mungbean and other legumes, which reported enhanced osmolyte (proline and GB) accumulation and antioxidant enzymes (SOD, POD, CAT) activity but reduced Pn under drought (Farooq et al., 2009; Mandi et al., 2018; Iqbal et al., 2019; Kumar et al., 2021; Nair et al., 2021).

The heatmap (**Figure 6**) rectified these patterns, indicating co-expression of physiological and biochemical traits in tolerant genotypes, especially under water scarce conditions. Trait clustering reflected the stimulation of combined drought-responsive pathways, in agreement with transcriptomic analyses by Singh et al. (2021) and Azam et al. (2023). Moreover, **Figure 6**, which illustrated the hierarchical clustering of mungbean genotypes based on overall trait performance, showed that drought stress significantly enhanced genotypic differences, making tolerant genotypes easily distinguishable from susceptible ones. Similar clustering patterns under drought treatments were also reported in topical studies on legumes, where multivariate classification was used to discern the stress-tolerant genotypes (Ghanbari and Javan, 2015; Azam et al., 2023).

The consistency across physiological and biochemical parameters was further confirmed at the molecular level. Expression analysis of key drought-responsive genes showed a clear upregulation in tolerant genotypes (**Figure 7**). Genes involved in osmotic regulation (*VrP5CS1* and *VrBADH*), oxidative defense (*VrSOD1*, *VrCAT1*, *VrPOD1*), and water transport (*VrPIP2-1*) were significantly over-expressed in tolerant mungbean genotypes, particularly ‘BARI Mung-8’ and ‘K851’. Transcription factors like *VrDREB2A* and late embryogenesis abundant protein gene *VrLEA3* were also markedly upregulated, validating the role of ABA-independent stress signaling in regulating downstream protective responses. These results were supported by Alsamadany (2022); Alzahrani (2024) and Alghabari and Shah (2024), who reported that drought tolerance is closely tied to the transcriptional regulation of antioxidant pathways and osmolyte biosynthesis in different crops under different kinds of abiotic stresses. The aquaporin gene *VrPIP2-1*, which regulate water uptake and intracellular movement, depicted the highest expression in ‘BARI Mung-8’ and ‘L-92’, corresponding to their higher RWC (**Figure 1**), thereby providing a molecular reason for physiological water retention. Moreover, *VrCHLH*, involved in chl biosynthesis, remained stable in tolerant mungbean genotypes but declined in susceptible ones, aligning with chlorophyll data presented in **Figure 1** and authenticated the findings by Al-Dulaimi et al. (2022) and Ariharasutharsan et al. (2022).

Altogether, the combined and integrated evaluation across **Figures 1**-**7** confirmed that drought tolerance in mungbean is not determined by isolated traits, but by a concerted interaction of physiological, biochemical, and molecular mechanisms. The strong overlap between molecular expression patterns and physio-chemical traits explicates that tolerant genotype such as ‘BARI Mung-8’, ‘BMX-010015’, ‘K851’, and ‘L-92’ possess coordinated stress defense networks that nullify the impact of drought. These genotypes not only showed high trait stability under stress but also strong transcriptional activation of key stress-responsive genes, making them ideal candidates for climate-resilient breeding. This work provides a dynamic platform for integrating trait selection, transcript profiling, and marker-assisted screening in mungbean improvement programs. The multi-level coherence of responses across physiological, biochemical, and gene expression explains the complex but predictable architecture of drought tolerance and supports the adoption of integrative phenotyping and genotyping frameworks for legume crop stress resilience under changing climatic conditions.

## Data Availability

The original contributions presented in the study are included in the article/supplementary material. Further inquiries can be directed to the corresponding author.
